# Transcending time: the forensic anthropological case study of three unidentified transgender women in Italy in the early 1990s

**DOI:** 10.1007/s00414-023-03122-x

**Published:** 2023-11-15

**Authors:** Andrea Palamenghi, Lorenzo Franceschetti, Stefano Tambuzzi, Annalisa D’Apuzzo, Debora Mazzarelli, Cristina Cattaneo

**Affiliations:** LABANOF, Laboratorio Di Antropologia E Odontologia ForenseSezione Di Medicina Legale, Dipartimento Di Scienze Biomediche Per La Salute, Università Degli Studi Di Milano, Via Luigi Mangiagalli 37, 20133 Milan, Italy

**Keywords:** Forensic identification, Transgender, Gender-affirming surgeries, Missing persons, Skeletal remains

## Abstract

In forensic anthropology, the estimation of skeletal sex results in a dichotomous outcome: male or female. This poses challenges when dealing with transgender individuals. Italy has the highest rate per capita of transgender murders, making their identification a compelling issue. This study presents three cases of unidentified skeletal remains of transgender women (MtF) that underwent autopsies in the 1990s at the Institute of Legal Medicine of Milan. The transgender status of the individuals was determined from the autopsy records which indicated the co-existence of breast implants and male genitalia. The biological profile was created by estimating sex, age, ancestry, and stature, and the bones were investigated looking for evidence of Facial Feminization Surgery (FFS). Anthropological estimations revealed that the skeletons belong to male, adults, European individuals, between 164 and 184 cm. Fragmented gelatinous masses were found in association with two skeletons, which were interpreted as remnants of breast implants. In two cases, signs of remodeling of the cortical surface were observed on the zygomatic bones, although the bone marks observed here were not specific enough to link them to FFS. Despite some limitations, this study highlights the need for greater awareness among practitioners about this limitedly addressed issue, advocating for a more inclusive forensic anthropology that strives to improve methods and interpretation of evidence for the identification of transgender individuals.

## Introduction

Biological sex is determined at conception and dictates dimorphic morphometric traits, while gender is a social construct tied to self-perception and identity. Gender dysphoria (GD) is the incongruence between the gender identity and the biological sex of an individual [1]. People experiencing GD are broadly referred to as transgender, and they may grapple with severe psychological and emotional distress and uneasiness with themselves, due to this disconnection and the subsequent feeling of internal and social rejection such as transphobia [2, 3]. Transgender individuals may perform a transition from male to female (MtF) or from females to males (FtM), opting for surgical treatment like Gender Confirmation Surgery (GCS). In particular, MtF women can choose to modify masculine facial features via Facial Feminization Surgery (FFS) [4, 5].

At present, only biological sex can be inferred from bone analysis [6], leaving out the determination of gender identities which cannot be reliably inferred solely from associated evidence, such as clothing or personal items [5]. Despite these limitations, the issue is paramount, especially in the light of the spreading of violence targeting trans and gender-diverse individuals with a reported 327 murders in 2022 alone.^1^ According to the Trans Murder Monitoring report from 2008 to 2022, Italy has the highest rate per capita of transgender murders. A 10-year retrospective study presented 20 cases of homicide of transgender individuals handled by the Institute of Legal Medicine of Milan, Italy [7]. Most victims were sex workers from South America, with three being native Italians. Signs of overkill and postmortem symbolical manipulation were found, stressing the brutality perpetrated by the offenders toward these victims.

In cases where soft tissues are preserved and have not been extensively damaged by postmortem decay, the possible presence of external genital organs and breast implants, or signs of mastectomy, coupled with circumstantial evidence, may suggest the individual’s transgender status. However, for highly decomposed or skeletonized remains, such evidence is often absent. This challenges forensic anthropologists to consider the possibility that the remains may belong to individuals who did not identify with their birth-assigned sex. When a discrepancy is noticed within a body or between anatomical and material evidence, it is essential to record and share this information with investigators. Such details can aid in refining potential identity matches and support the identification process. However, a recent survey indicates that some practitioners are reluctant to report such findings, deeming it beyond their scope [6].

By presenting the postmortem examinations and anthropological analyses of the remains of three unidentified transgender women from the Institute of Legal Medicine of Milan (Italy), this report aims at: (i) presenting the challenging task of determining the transgender status from skeletal remains; (ii) comparing the outcome of postmortem and anthropological examinations; (iii) providing additional evidence that could guide the identification and interpretation of bone signs possibly related to GCS; (iv) increasing practitioners’ awareness of this issue.

## Materials and methods

Three skeletons of transgender individuals that died between 1992 and 1996 and underwent post-mortem examinations at the Institute of Legal Medicine of Milan were analyzed. The transgender status of the individuals was derived from the autopsy records. As the investigations did not lead to personal identification, the bodies were then buried as unidentified individuals in cemeteries across the city of Milan.

According to the Italian cemetery policies, after ten years from burial, unclaimed human remains are to be disposed of in cumulative ossuaries. In order to avoid this, the Laboratory of Forensic Anthropology and Odontology (*Laboratorio di Antropologia e Odontologia Forense*—LABANOF) of the University of Milan retrieves these remains for identification purposes [8]. This activity aligns with Article 43 of the Presidential Decree of the Italian Republic (DPR) n.285 of September 10th, 1990, of the National Police Mortuary Regulation, and the accord between LABANOF and the University of Milan, and the special Commissioner of the Government for Missing Persons. Consequently, after complete skeletonization, the skeletal remains were exhumed between 2016 and 2019 and transferred to laboratory facilities in zinc boxes. The recovered remains presented different taphonomic patterns of preservation both in terms of quantity and quality of the remains. The completeness of the skeletons was assessed following the Bone Representation Index (BRI) which calculates the ratio between the number of bones recovered and the total number that should be anatomically present at the time of recovery [9].

The remains were laid down, and the biological profile was created, including ancestry [10, 11], sex [12–14], age [15, 16], and stature [17] estimations. Given that some skeletons were missing portions (e.g., pubic symphysis) due to postmortem damage, different methods were used based on the available skeletal material. Moreover, the bones were examined looking for evidence of gender-confirmation surgery (GCS) and facial feminization surgery (FFS), in accordance with the available literature [4–6, 18, 19].

## Results

For each case, the results of the post-mortem examination and anthropological study are reported below. Tables 1 and 2 summarize the postmortem examination findings and the biological profile of the three skeletons, respectively.

**Table 1:** Information from autopsy reports

Case	Sex	Ancestry estimation	Age-at-death estimation	Stature	Summary of external findings	Summary of autopsy findings	Cause of death
1	Male genitalia	Black	30–35 years	183 cm	- Female clothes - Earring holes - Female breasts with surgical scars	Breast implants	Overdose
2	Male genitalia	White	25–30 years	182 cm	- Female breasts with surgical scars	Breast implants	Undetermined
3	Male genitalia	Austramelanesoid	30–35 years	170 cm	- Female clothes and personal effects - Earring holes - Make-up and nail polish (hands and feet) - Ring on the 4^th^ right finger - Surgical scars on the face (right parotid and left mandibular regions) - Female breasts with surgical scars	Breast implants	Overdose

**Table 2:** Biological profile of the skeletons

Case	Skeletal sex estimation (12–14)	Ancestry estimation (10,11)	Age-at-death estimation (15,16)	Stature (17)	BRI (9)	Bone signs
1	Male	European	35–44 years	169–179 cm	5 (85%)	Medially curved zygomatic bones; porotic area on left zygomatic
2	Male	European	23–57 years	175–181 cm	4 (65%)	None
3	Male	European	21–30 years	163–169 cm	4 (65%)	Porotic and dense area on right zygomatic; porotic area on left zygomatic

*BRI* Bone Representation Index (9)

### Case 1

Case 1 was a 30/35-year-old black male (length 183 cm and weight 70 kg). The body was reported to be wearing a black bodysuit and blue pants. Earring holes were noted on the earlobes bilaterally. Bilateral breast prosthesis with associated scars were present. The external genitalia were male. The cause of death was overdose.

**Fig. 1:**
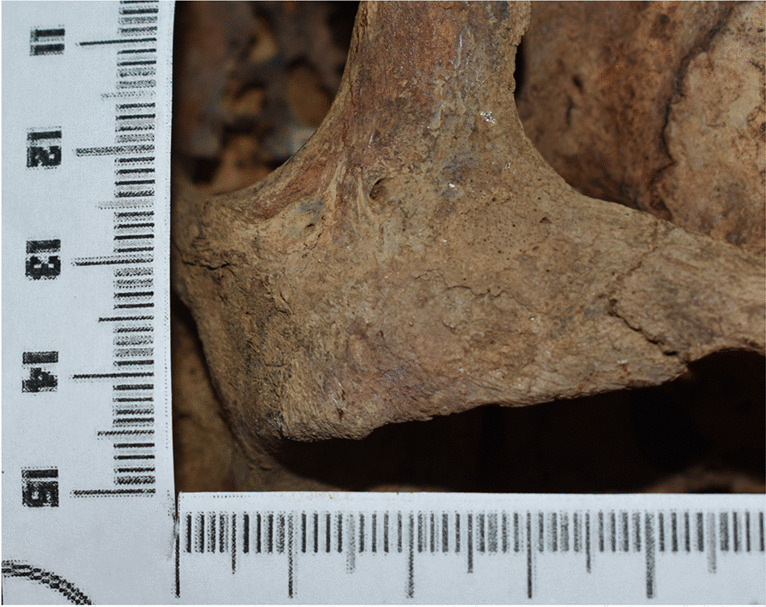
Bone apposition area on the left zygoma of skeleton of case 1

Skeleton 1 was estimated to be a European, male individual, aged between 23 and 57 years. The zygomatic bones present as medially curved toward the infratemporal fossa. On the posterior third of the left zygomatic bone, a circular area (11 mm of diameter) with deposition of porous bone was found (Fig. 1). The surrounding surface was compromised with exfoliation of the cortical bone due to postmortem damage, hampering a thorough evaluation.

### Case 2

Case 2 was a 25/30-year-old white male (length 182 cm and weight 74 kg). The body had no visible decomposition features. The body was naked as it came from a city hospital. Bilateral breast prosthesis with associated scars was observed. The external genitalia were male. Breast implants were found bilaterally at autopsy dissection. The cause of death was undetermined.

Skeleton 2 was estimated to be a European, male individual, aged between 35 and 44 years. This skeleton did not present any macroscopic or microscopic alterations of the cortical bone affecting the cranium. No sign that could be associated with feminization surgery was found. Several small fragments of gelatinous silicone-like material, embedded in soil, were recovered when sieving the burial soil.

### Case 3

Case 3 was a 30/35-year-old male (length 170 cm and weight 67 kg). The body had no visible decomposition features. The ancestry was described as Austromelanesoid, according to the classification available at the time of the autopsy. The body was reported to be wearing a wig, with approximately 20-cm-long brown-reddish hair, several hairpins, and a leopard-type clip. Other garments included a scarf around the neck, a black corset, white pants, a black panty girdle, black leathery belts legging, black hold-up stockings, gray socks and leathery stiletto boots with metal heels. Make-up was found on the face, and earring holes were noted on the earlobes bilaterally. The victim was wearing a ring on the fourth finger of the right hand. Pink and red polish on the nails of hands and feet respectively was noted. On the face, two flat surgical scars were described in the right parotid and in the left mandibular regions. Bilateral breast prosthesis with associated scars was found. The external genitalia were male. The cause of death was overdose.

**Fig. 2:**
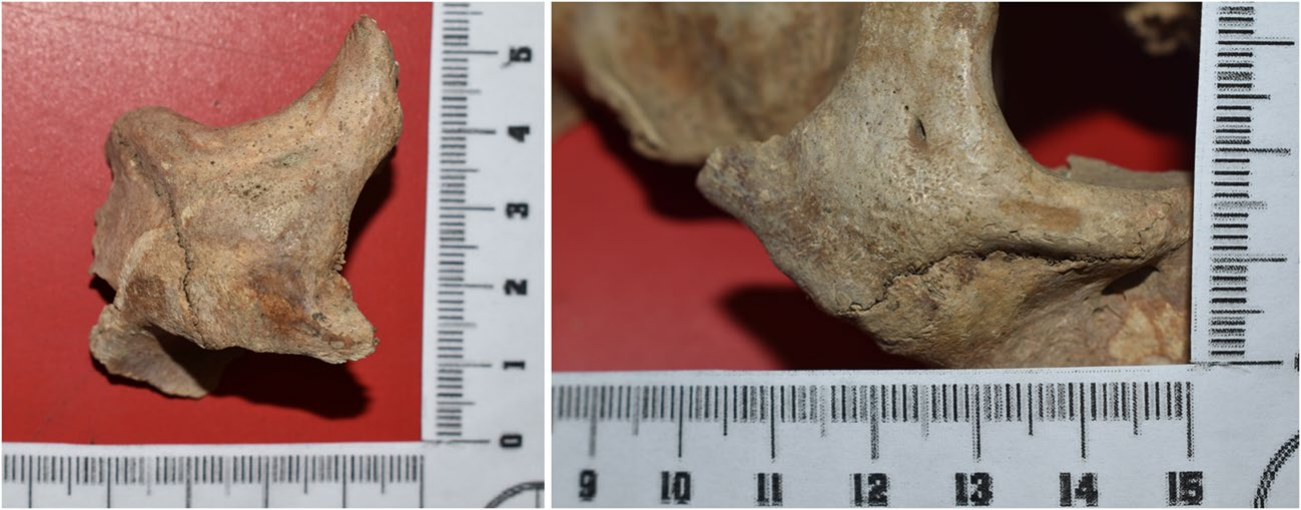
Dense areas on the right (right panel) and on the left (left panel) zygoma of skeleton of case 3

**Fig. 3:**
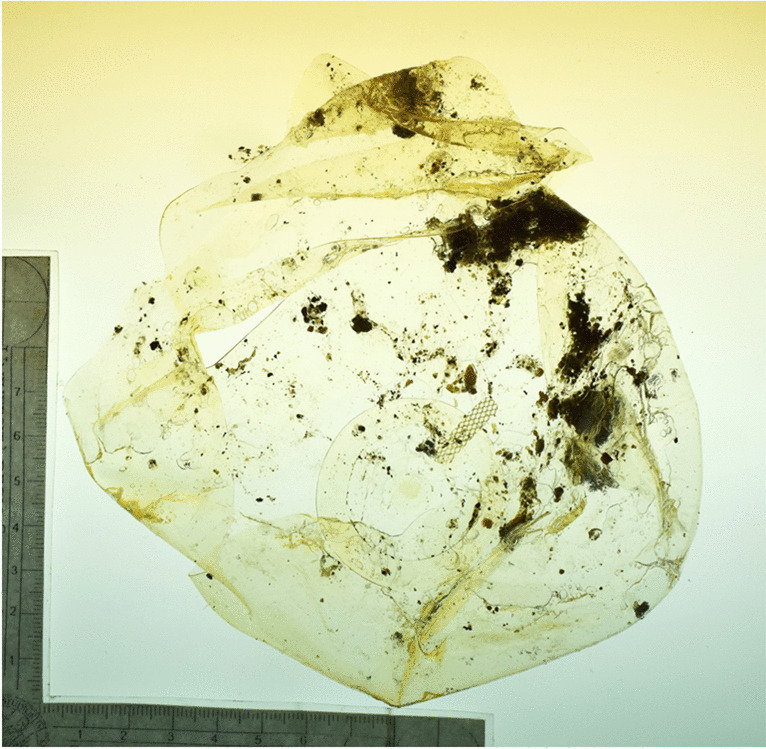
Breast prosthesis of case 3

Skeleton 3 was estimated to be a European, male individual, aged between 21 and 30 years. The extensive fragmentation of the cranium hampered a comprehensive analysis both for the biological profile and the detection of feminization signs. In its middle third, the right zygomatic bone presented a compact bone area, measuring 17 mm on a horizontal axis and 15 mm on a vertical axis, surrounded by porotic cortical bone. The bone here was remodeled, highly dense, possibly meaning that the timing of remodeling was not recent. On the left zygomatic bone, in its middle third, a porotic circular area extended for 13 mm on a horizontal axis and 11 mm on a vertical axis, with evidence of macro and microporosity. Figure 2 displays the relevant skeletal findings. A breast prosthesis (Fig. 3) was found after examining and sieving the burial soil.

## Discussion

In forensic anthropology, the identification of remains that might belong to transgender individuals is not commonplace, and such a circumstance might not always be given adequate consideration [6, 20]. Forensic anthropologists have only recently begun to grapple with the binary nature of sex assessment, advocating for a more inclusive methodology for sex estimation that encompasses the variability found in transgender individuals [5, 6, 20, 21]. This estimation can be challenging, as solely the skeletal features can be evaluated, and no reliable tool is available to assume a gender identity [5, 6]. As expected, in this study, the skeletal sex estimation based on morphological evaluation of the cranium and the pelvis classified the individuals as males. Thus, inferring transgender status based solely on skeletal evidence was not feasible. This has some serious repercussions when attempts are made to identify skeletal remains, especially in the absence of other evidence.

From a practical standpoint, there is the relevant issue of how transgender individuals are classified within the registers of missing persons and of unidentified corpses [20]. In the registry of missing persons, a transgender person should be listed under their self-identified gender, as this reflects their lived identity and is typically the gender under which their disappearance would have been reported. This respects of course the individual’s affirmed identity but may not necessarily be able to effectively assist in making a successful match with unidentified remains. In fact, there may be the case of a corpse found in an advanced state of decomposition or skeletonized, on which it is not possible to make an adequate assessment of the skin surface and soft tissue. Thus, there could be a transgender individual who is classified according to the biological sex and then entered in the wrong database, significantly reducing the chances of achieving proper identification. Notably, in the three cases studied, no clothing or other items indicative/suggestive of transgender identity were found when only skeletal remains were examined.

The question then arises: how can anthropologists gather skeletal and associated evidence that would support the hypothesis of the victims being transgender, so that the transition does not lessen the possibility of being identified [5, 21]?

This case series did not identify specific skeletal indicators, but supportive findings were observed. For example, in two cases fragments of gelatinous material were recovered embedded in the burial soil of each skeleton, after accurate sieving procedures. Such a finding highlights the importance of thorough recovery and examination of residues in the soil which may provide valuable insights to the investigations [22, 23].

After careful assessments, they were interpreted as possibly coming from the decomposition of breast implants, which is consistent with these individuals’ autopsy report describing their presence. In one case, however, no such gelatinous material was found, although the deceased had breast implants. Clothing or other items offer limited reliability in such cases [5], as they can be dislodged during skeletonization and lost, especially, when the body is in an outdoor environment. On the contrary, the presence of secondary identifiers or circumstantial evidence can serve as additional indicators, which could raise the suspicion of a transgender individual, given the limitations of skeletal analysis alone.

MtF individuals may undergo surgical procedures like facial feminization surgery (FFS) reconstruction, which heavily involves modifications of the maxillo-facial skeleton [4, 6]. Common sites of modification include the glabellar region and the mandible [4]. However, no modification altering the original morphology of the glabella or mental regions was observed in the skeletons of this study.

Other anatomical portions considered in FFS involve the nose, hairline, and cheekbones: however, these changes may not leave evidence on the skeleton [5, 6], so they are not always easily recognizable. In this series, the only anomalies observed included small porotic and remodeled areas on the zygomatic bones in two cases. The literature on FFS mentions the zygomatic bones for injection of fillers of placement of implants for cheek augmentation [4, 18]. Enhancement of the malar projection may also include the creation of a defect in the zygomatic bone through osteotomy and placement of a bone graft [19]. The postmortem report of case 3 detailed two bilateral scars in the right and left parotid regions, which arguably are the results of a surgical procedure, and bilateral anomalies of the cortical surface were observed on the skeletal remains. In case 1, signs were observed only on one side, which is not consistent with FFS procedures. Therefore, considering the dearth of detailed reports, in addition to the discordant evidence collected, we are far from confidently confirming or ruling out that these signs are related to FFS, based only on the skeletal evidence. The bone marks observed here are not specific enough to link them to FFS procedures. Gender confirming surgical procedures were pioneered in the USA in the 1980s and 1990s [4], and the three cases represent victims from the early to mid 1990s in Italy. Possibly, FFS procedures at that time were not as widespread as they are today. Arguably, these women did not undergo or could not access FFS.

Although a study on a limited sample indicated that pelvic measurements may reveal a shift toward a male metric configuration in FtM individuals [24] and while there is variable evidence regarding the interplay between hormone therapies and bone tissue, how gender transition affects the skeleton is still poorly understood [6, 25]. More research is needed to better understand the effects of hormone replacement therapy and gender confirmation surgeries on the skeleton. Investigating specific changes in bone density, structure, and growth resulting from these treatments could potentially reveal identifiable markers for transgender individuals. Nevertheless, these procedures do not seem to significantly affect the parameters used in standard sex estimation methods. A recent application of Fordisc for craniometric sex estimation on CT-scans of MtF transgender individuals concluded that post-surgery, metric methods would most likely still determine the individual biological sex, albeit with reduced probabilities and typicalities [5, 21].

The anthropological examination on the remains did not produce any definitive skeletal evidence that could be confidently linked to FFS. However, circumstantial evidence and thorough recovery procedures arguably support the hypothesis of the transgender status. When properly recovered, these elements, could possibly serve as red flags for forensic anthropologists who are aware of the challenging issue of identifying transgender individuals.

Transgender women and men encompass a diverse population with different transition journeys and medical histories which add to the complexity. While acknowledging certain limitations, this study offered a significant contribution to this cause, highlighting the significance of a comprehensive and multifaceted approach to the intricacies of gender identity within the forensic identification process. In addition to adopting a multidimensional forensic approach in such scenarios, it is imperative to maintain an inclusive perspective that takes these situations into account, especially in light of the escalating violence targeting this part of the society.

## Data Availability

The data that support the findings of this study are not openly available due to reasons of sensitivity and are available from the corresponding author upon reasonable request. Data are located in controlled access data storage at LABANOF, University of Milan.

## Footnotes

1 https://transrespect.org/en/map/trans-murder-monitoring/
